# Covert Brain Infarcts in Patients with Philadelphia Chromosome-Negative Myeloproliferative Disorders

**DOI:** 10.3390/jcm11010013

**Published:** 2021-12-21

**Authors:** Polina I. Kuznetsova, Anton A. Raskurazhev, Rodion N. Konovalov, Marina V. Krotenkova, Andrey O. Chechetkin, Olga V. Lagoda, Anait L. Melikhyan, Marine M. Tanashyan

**Affiliations:** 1 Department of Angioneurology, Research Center of Neurology, 125367 Moscow, Russia; rasckey@live.com (A.A.R.); angionev@gmail.com (O.V.L.); M_Tanashyan2004@mail.ru (M.M.T.); 2 Neuroimaging Department, Research Center of Neurology, 125367 Moscow, Russia; krn_74@mail.ru (R.N.K.); krotenkova_mrt@mail.ru (M.V.K.); 3 Ultrasound Diagnostics Department, Research Center of Neurology, 125367 Moscow, Russia; andreychechetkin@gmail.com; 4 Department of Standardization and Treatment Methods, National Research Center for Hematology, 125167 Moscow, Russia; anoblood@mail.ru

**Keywords:** Philadelphia chromosome-negative myeloproliferative disorders, covert brain infarcts, hematology, endothelial dysfunction

## Abstract

Backgrounds and Purpose. Philadelphia chromosome-negative myeloproliferative disorders (Ph-negative MPD) are a rare group of hematological diseases, including three distinct pathologies: essential thrombocythemia (ET), polycythemia vera (PV), and primary myelofibrosis (PMF). They most often manifest with thrombotic complications, including cerebrovascular events. Covert brain infarcts (CBIs) are defin ed as predominantly small ischemic cerebral lesions that are detected using magnetic resonance imaging (MRI) in the absence of clinical stroke events. The relationship between MPD and CBIs remains unclear. Methods. Included in the study were 103 patients with the diagnosis of Ph-MPD (according to WHO 2016 criteria) (median age—47 (35; 54) years; 67% female). In total, 38 patients had ET, 42 had PV, and 23 had PMF. They underwent clinical examination, routine laboratory analyses (complete blood count), brain MRI, ultrasound carotid artery, flow-mediated dilatation (as a measure of endothelial dysfunction—FMD). Results. Overall, 23 patients experienced an ischemic stroke (as per MRI and/or clinical history), of which 16 (15.5%) could be classified as CBIs. The rate of CBIs per MPD subtype was statistically non-significant between groups (*p* = 0.35): ET–13.2%, PV–21.4%, and PMF–8.7%. The major vascular risk factors, including arterial hypertension, carotid atherosclerosis, and prior venous thrombosis, were not associated with CBIs (*p* > 0.05). Age was significantly higher in patients with CBIs compared to patients without MRI ischemic lesions: 50 (43; 57) years vs. 36 (29; 48) (*p* = 0.002). The frequency of headaches was comparable between the two groups. CBIs were associated with endothelial dysfunction (OR - 0.71 (95% CI: 0.49–0.90; *p* = 0.02)) and higher hemoglobin levels (OR—1.21 (95% CI: 1.06–1.55); *p* =0.03). Conclusions. CBIs are common in patients with Ph-negative MPD. Arterial hypertension and carotid atherosclerosis were not associated with CBIs in this group of patients. The most significant factors in the development of CBIs were endothelial dysfunction (as measured by FMD) and high hemoglobin levels. Patients with Ph-negative MPD and CBIs were older and had more prevalent endothelial dysfunction.

## 1. Introduction

Cerebrovascular pathology remains one of the most widespread and socially significant diseases worldwide. Silent cerebrovascular disease is common and associated with future risk for stroke and dementia [1]. Covert brain infarcts (CBIs) are small ischemic cerebral lesions that can be detected using MRI without stroke events [2,3]. It is known that a higher prevalence of CBIs has been associated with aging and common vascular risk factors such as arterial hypertension, carotid atherosclerosis, atrial fibrillation. This was shown in The Cardiovascular Health Study (CHS) (population-based, longitudinal study of 5888 people aged 65 years or more); on follow-up MRI, 254 out of 1433 participants (17.7%) had one or more CBI [4]. The latter may arise as a result of endothelial dysfunction (e.g., arteriolar wall thickening, local thrombosis, etc.) [5]. It has been previously demonstrated that endothelial dysfunction is one of the earliest indicators of cardiovascular disease and is associated with CBIs. Flow-mediated dilation (FMD) is the most widely used method to study endothelial function [6,7].

Blood pathology is often associated with endothelial dysfunction and cerebrovascular disease. Philadelphia chromosome-negative myeloproliferative disorders (Ph-negative MPD) are a rare group of blood diseases that include polycythemia vera (PV), essential thrombocythemia (ET), and primary (idiopathic) myelofibrosis (PMF) [8]. They are characterized by the proliferation of all myelopoiesis stem cells with a high level of differentiation strongly associated with Janus kinase 2 (JAK2), calreticulin (CALR), and myeloproliferative leukemia virus oncogene mutations [9]. The main complications include thrombotic (including stroke) and hemorrhagic events, which may contribute significantly to mortality. Worldwide incidence and prevalence data are inconclusive, but based on U.S. data the incidence of PV and ET is 1.0–2.0 per 100,000 person years, while PMF is rarer with an incidence of 0.3 per 100,000 person years [10].

Epidemiological data on cerebrovascular pathology in Ph-negative MPD are sparse and usually deal with symptomatic stroke. In this study, we aimed to evaluate the prevalence of CBIs in this clinical setting and their possible association with FMD as a measure of endothelial dysfunction.

## 2. Materials and Methods

**Figure 1:**
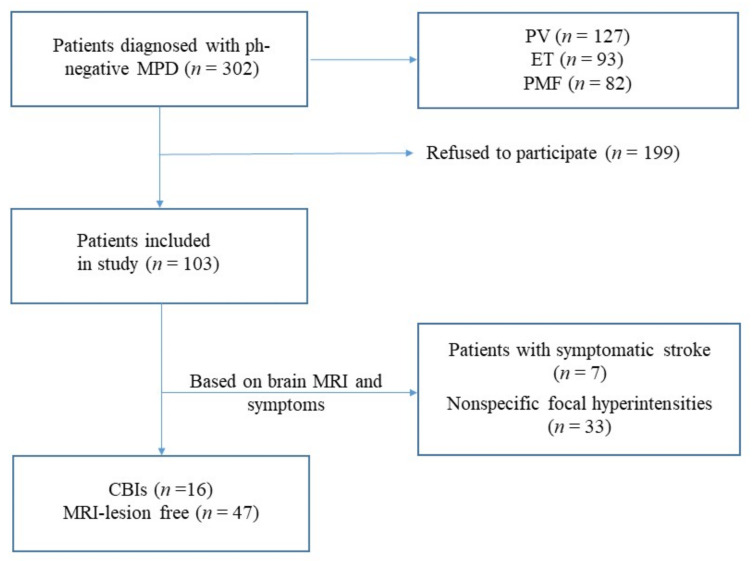
Patient selection flowchart.

This was a cross-sectional study of the prevalence of neurological symptoms in patients with Ph-negative MPD. The study lasted from May 2014 till January 2016. All patients who were diagnosed with Ph-negative MPD during this period (according to WHO 2016 criteria) at the National Research Center for Hematology (department of standardization and treatment methods), Moscow, Russia, were then referred to the Research Center of Neurology, Moscow, Russia, regardless of symptoms (Figure 1). A total of 103 patients (median age—47 (35; 54) years, 67% female) provided informed consent and comprised the study group: 38 patients with ET, 42–PV, 23–PMF. At the study site (RCN) they underwent a thorough clinical examination (including risk stratification according to NCCN, DIPSS, and IPSET for PV, PMF, and ET, respectively), routine laboratory analyses (including complete blood count), brain MRI, carotid artery ultrasound, flow-mediated dilation (FMD) and were screened with transthoracic echocardiography (there were no indirect signs of the presence of patent foramen ovale).

**Figure 2:**
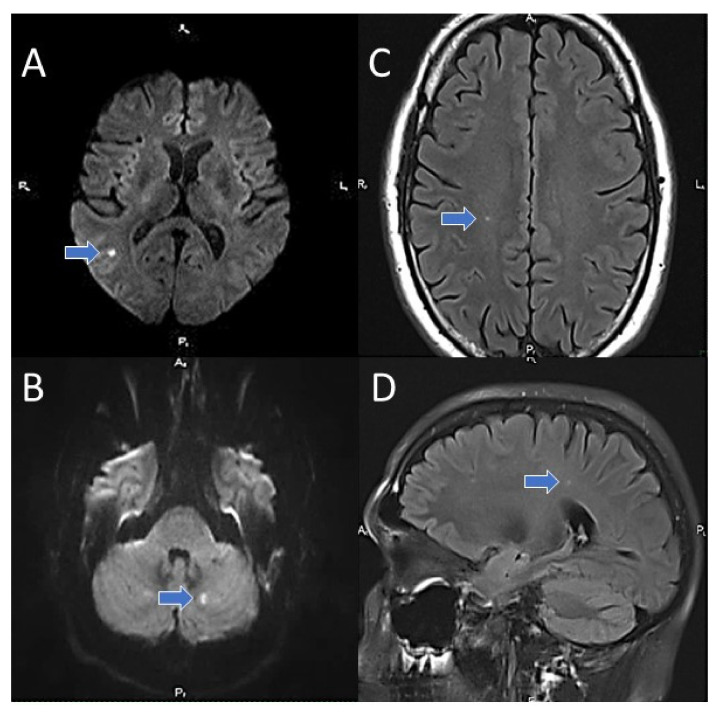
Covert brain infarcts in patients of the study group (MRI). (**A**,**B**)–DWI; (**C**,**D**)–T2 FLAIR.

The presence, localization and size of focal lesions in the brain were investigated using magnetic resonance imaging (MRI). MRI was performed in standard modes using T2, T1, T2 FLAIR, and diffusion weight imaging—DWI. The criterion for assigning a patient to the covert brain infarcts group was any clinically silent focus of restricted diffusion (high DWI signal and low ADC value) occurring either white or gray matter, located in the cerebrum, cerebellum, or brain stem (Figure 2). MRI scanning was performed on a Magnetom Verio SIEMENS tomograph (Germany) with a magnetic induction of 3 Tesla.

The state of carotid arteries was examined using duplex scanning on a Toshiba Viamo device, Japan iU 22 (Phillips). In this case, the thickness of the “intima-media” layer up to 1 mm was taken as the norm; more than 1 mm was regarded as pathological. A local change in the vessel wall, containing inclusions of increased intensity of the ultrasound signal, and/or the detection of a vessel wall thickening of more than 1.5 mm was the basis for the diagnosis of atherosclerotic plaque. The vasomotor function of the endothelium was assessed using ultrasound according to the method of D. Celermajer (1992) with the study of flow-mediated dilation (FMD) of the brachial artery [11]. The study was approved by the local ethics committee; all patients provided written informed consent.

### Statistics

Statistical analysis was performed in R (packages “dplyr” and “ggplot2”), version 4.0.5. The following non-parametric methods were used: median and interquartile range for descriptive statistics, Mann–Whitney U test when comparing two independent samples, 2-sample test for equality of proportions with continuity correction, 3-sample test for equality of proportions without continuity, and Kruskal–Wallis rank sum test (when comparing 3 independent samples). A multivariable logistic regression was performed. All statistical tests were two sided and were performed with an alpha level of 0.05. The null hypothesis was rejected if *p* < 0.05.

## 3. Results

### 3.1. Patient Characteristics

The main demographic and clinical characteristics of patients in the study group are presented in Table 1. The median age of patients with MPD was 40 (30.5; 52.5) years for ET, 51 (41.2; 55) years for PV, and 46 (33.5; 53) years for PMF.

The prevalence of the main vascular risk factors in patients with MPD, such as arterial hypertension, carotid atherosclerosis, venous thrombosis, diabetes mellitus type 2, myocardial infarction, stroke, presence of the JAK2 V617F mutation, antiplatelet therapy status (acetylsalicylic acid) and frequency of headaches, is presented in Table 1.

### 3.2. Prevalence of CBIs

Two groups were then identified from our cohort of patients: CBIs patients (*n* = 16) and those without any lesions on MRI (*n* = 47). Patients with clinically symptomatic stroke (*n* = 7) were not included in further analysis.

The major vascular risk factors, including arterial hypertension, carotid atherosclerosis, and prior venous thromboses, were not associated with CBIs (*p* >0.05). Age was significantly higher in patients with CBIs compared to patients without MRI ischemic lesions: 50 (43; 57) years vs. 36 (29; 48) (*p* < 0.05), which is consistent with previous research [12]. The frequency of headache was comparable between two groups.

No difference in the prevalence of CBIs among different subtypes of MPD was observed: 5 (13.2%), 9 (21.4%), and 2 (8.7%) patients in ET, PV, and PMF subgroups, respectively (*p* = 0.35). CBIs were associated with endothelial dysfunction (via FMD: 5.5 (4.0; 8.0) % vs. 11 (7; 13) %; *p* < 0.01) and higher hemoglobin levels (151 (140.5; 162) g/l vs. 135 (121; 144) g/l; *p* = 0.01). A lower platelet count was observed in patients with CBIs—433 (268; 517) × 10^9^ vs. 552 (465; 619.5) × 10^9^, *p* < 0.05 (Table 2).

**Figure 3:**
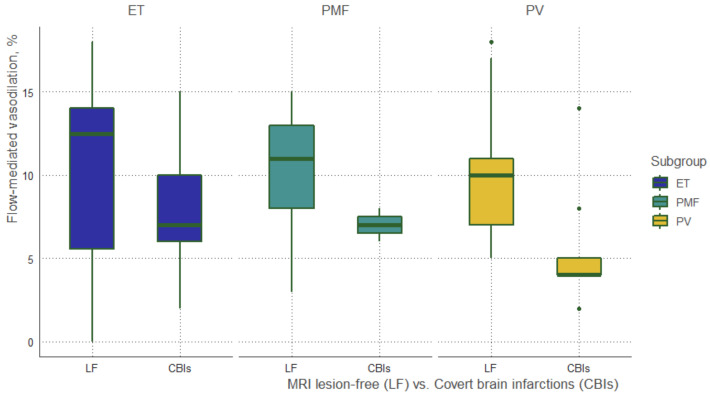
Flow-mediated dilation (FMD) rates. MRI lesion-free vs. CBIs.

The lowest flow-mediated dilation (FMD) rates were observed in patients with CBIs among all subtypes of MPD: 7% (6;10) vs. 12.5% (5.6; 14.0) in ET, 7.0% (6.5; 7.5) vs. 11% (8;13) in PMF and 4% (4;5) vs. 10% (7;11), respectively (Figure 3).

### 3.3. Multivariable Analysis

We performed a multivariable logistic regression (MLR) analysis with all variables from Table 2 to elucidate their possible association with CBIs (Table 3). The OR for FMD was 0.71 (95% CI: 0.49, 0.90, *p* = 0.02), and the OR for hemoglobin level was 1.21 (95% CI: 1.06, 1.55; *p* = 0.03), indicating that lower FMD and higher hemoglobin levels were associated with CBIs. The presence of headaches was borderline significant with an OR of 0.04 (95% CI: 0.0006, 0.60; *p* = 0.05).

## 4. Discussion

Covert brain infarcts are a frequent incidental finding on brain images [13] and have a prevalence of 10% to 30% in healthy elderly populations, and 30% to 50% in populations with elevated cardiovascular risk [14]. Age, arterial hypertension, atrial fibrillation, and large-artery atherosclerosis are among the most strongly associated with CBIs risk factors, along with cardiovascular surgery. However, the epidemiology of CBIs in the setting of Ph-negative myeloproliferative disorders has not been established.

In our study group, patients with CBIs were significantly older (50 (43; 57) vs. 36 (29; 48) years; *p* < 0.01), which is unsurprising given the association of CBIs with age [15]. The prevalence of CBIs was 15.5%, which may be higher compared to population-based studies with the mean age at or around 50 years: out of seven publications with a mean age of less than 55 years, five found CBIs in 5.2–5.8% (total number of patients ≈ 6,300), the remaining two found CBIs in 20.8% (*n* = 476) and 32.5% (*n* = 1008) [13]. This may possibly indicate that patients with Ph-negative MPD develop CBIs at a younger age than in the general population, yet a relatively small sample size (103 patients) does not allow one to establish a clear relationship.

Conventional stroke risk factors, such as hypertension and carotid atherosclerosis, were similarly distributed among patients with and without CBIs. The prevalence of arterial hypertension was ≈20% in both groups, which is lower than in several previous studies (64% in patients with PV and ET in [16]; 52.3% in PV patients in a study by Bevevolo et al. [17]), which in part may be attributed to a younger age. The latter study [17] also found hypertension to be associated with increased thrombotic risk (hazard ratio (HR)—1.77 (95% CI: 1.03–3.06; *p* = 0.04). Carotid atherosclerosis was non-significantly more prevalent in patients with CBIs, and according to limited data on ET patients [18], no significant differences were found in carotid artery atherosclerosis vs. the control group.

Prior venous thrombosis (VT) serves as a risk factor for future cardiovascular events in patients with Ph-negative MPD, especially in the setting of ET (based on the IPSET risk score). In our study, the rate of prior VT did not differ in patients with and without CBIs—3 (2 ET and 1 PV) and 5 (3 ET, 1 PV, and 1 PMF) patients, respectively [19].

The V617F mutation in the JAK2 gene is a hallmark of Ph-negative MPD—especially PV (where it is present in >90% of patients) and ET (half of the patients). In a recent publication by Košťál M. et al. [20], the JAK2 V617F mutation was a significant risk factor for stroke/TIA events (OR—2.303, 1.275–4.159; *p* < 0.01) in a cohort of patients with MPD. We did not find this association for CBIs in our study. A possible explanation for this may be the high overall proportion of JAK2 carriers in our group—almost 70%.

It is known that headaches may be an independent risk factor for the development of cerebrovascular disease in general and ischemic stroke in particular; however, in our study, such a relationship was not found [21,22]

According to the multivariable logistic regression model, the most significant factors associated with the development of CBIs were endothelial dysfunction (as defined by lower values of flow-mediated vasodilatation (FMD)) with an OR of 0.71 (95% CI: 0.49–0.90, *p* = 0.02) and hemoglobin levels (OR—1.21 (95% CI: 1.06–1.55); *p* = 0.03). It has been previously shown that endothelial dysfunction is common among patients with MPD, especially with thrombotic events [23,24]. FMD is a functional way to assess NO-mediated endothelial function; it involves measuring brachial artery dilation following a transient (≈5 min) period of forearm ischemia [25]. Lower FMD values are associated with endothelial dysfunction. We hypothesize that several factors in patients with Ph-negative MPD can change the “normal” endothelium, ultimately inducing changes that lead to CBIs. In particular, reactive oxygen species and intracellular proteases produced by activated neutrophils can damage endothelial cells and may lead to arteriolar wall thickening. Constantly circulating markers of endothelial activation, various selectins that propagate cell aggregation may lead to the development of thrombosis and CBIs among patients with MPD [24].

Elevated hemoglobin (Hb) concentration has been shown, in a large cohort of Scandinavian blood donors, to be associated with an increased risk of vascular events—e.g., for men, the levels of Hb 16.0–17.4 g/dL carried a risk of ischemic stroke with an HR of 1.55 (1.38–1.75) [26]. Kishimoto et al. [27] demonstrated that both low and high Hb levels were associated with endothelial dysfunction (as measured by FMD and nitroglycerin-induced vasodilation). This finding may in part explain the association found in our study between higher Hb concentration and CBIs.

The limitations of our study include a relatively small sample size, possible selection bias (since we included patients from only one clinical center and not everyone agreed to continue the examination at the neurological center), representation bias (not all patients diagnosed with Ph-negative MPD during the study period did participate in this study). Furthermore, we did not perform any additional diagnostic test to identify other possible causes of stroke (e.g., transesophageal echocardiography, prolonged electrocardiogram monitoring, etc.). Neuroimaging findings should be interpreted with caution as DWI-negative and FLAIR-positive small lesions might represent lesions caused by small vessels disease rather than definite covert brain infarcts. FMD, as a method of evaluating endothelial function, has significant limitations and requires further independent validation.

## 5. Summary

Covert brain infarcts are common in patients with Ph-negative MPD. Arterial hypertension and carotid atherosclerosis were not associated with CBIs in this group of patients. The most significant factors in the development of CBIs were endothelial dysfunction (as measured by FMD) and high hemoglobin levels. Patients with Ph-negative MPD and CBIs were older and had more prevalent endothelial dysfunction.

## Figures and Tables

**Table 1:** The prevalence of the main risk factors for stroke.

	Essential Thrombocytemia	Polycytemia Vera	Primary Myelofibrosis	MPD (All)	*p*
Number (%)	38 (36.9)	42 (40.77)	23 (22.33)	103 (100)	N/A
Age (median (1st quartile; 3rd quartile)	40 (30.5; 52.5)	51 (41.2; 55)	46 (33.5; 53)	47 (35; 54)	0.09
Female (%)	29 (76.3)	22 (52.4)	18 (78.3)	69 (67)	0.03
Arterial hypertenison (%)	9 (23.7)	14 (33.3)	7 (30.4)	30 (29.1)	0.63
Diabetes mellitus type 2 (%)	1 (2.6)	1 (2.4)	0 (0)	2 (1.9)	0.74
Myocardial infarction (%)	2 (5.3)	3 (7.1)	1 (4.3)	6 (5.8)	0.88
Venous thromboses (%)	7 (18.4)	6 (14.3)	1 (4.3)	14 (13.6)	0.29
Risk category					
Low (%)	12 (31.6)	23 (54.8)	20 (87.0)	55 (53.4)	<0.01
Intermediate (%)	18 (47.4)	12 (28.6)	3 (13.0)	33 (32.0)	0.02
High (%)	8 (21.0)	7 (16.6)	-	15 (14.6)	0.83
JAK V6717F carrier (%)	22 (57.9)	40 (95.2)	13 (56.5)	75 (72.8)	<0.01
Carotid atherosclerosis (%)	8 (21.1)	17 (40.5)	6 (26.1)	31 (30.1)	0.15
Antiplatelet therapy (%)	32 (84.2)	37 (88.1)	20 (86.9)	89 (86.4)	0.88
Headache (%)	25 (65.8)	25 (59.5)	18 (78.3)	68 (66)	0.31

**Table 2:** Covert brain infarcts compared to patients without MRI ischemic lesions.

	CBIs (*n* = 16)	MRI Lesion-Free (*n* = 47)	*p*
Age	50 (43; 57)	36 (29; 48)	<0.01
Arterial hypertension (%)	3 (18.75)	10 (21.3)	0.83
Atherosclerosis (%)	6 (37.5)	7 (14.9)	0.12
Venous thromboses (%)	3 (18.75)	5 (10.6)	0.68
JAK carriers	13 (81.25)	31 (65.9)	0.40
Female (%)	9 (56.25)	35 (74.5)	0.29
Male (%)	7 (43.75)	12 (25.5)	0.29
JAK2 (allele burden)	14.5 (5.7; 34.0)	6 (0; 16.5)	0.08
Headache (%)	9 (56.25)	32 (68.1)	0.58
Flow-mediated vasodilation	5.5 (4.0; 8.0)	11 (7;13)	<0.01
Hemoglobin, g/L	151 (140.5; 162)	135 (121; 144)	0.01
Erythrocytes	4.75(4.2; 5.0)	4.8 (4.2; 5.4)	0.75
Thrombocytes	433 (268; 517)	552 (465; 619.5)	<0.05
Leukocytes	7.4 (5.7; 8.9)	7.1 (5.9; 8.8)	0.84
ESR *	6 (5; 10.5)	5 (2.5; 10.5)	0.51
Hydroxyurea (%)	13 (81.3)	25 (53.2)	0.09

* Erythrocyte sedimentation rate.

**Table 3:** Multivariable logistic regression analysis.

	B	S.E.	O.R.	C.I (95%)		*p*
(Intercept)	−1.88	9.47	7 × 10^−9^	1 × 10^−18^	0.06	0.04
Age	0.12	0.08	1.12	0.97	1.38	0.16
AH+	−2.78	1.79	0.06	0.0005	1.27	0.12
AS	2.20	2.14	9.15	0.35	3 × 10^3^	0.30
VT	1.70	2.67	5.5	0.11	1 × 10^3^	0.45
JAK2 carrier	1.81	2.60	6.08	0.08	5.7 × 10^3^	0.49
Male	−0.56	1.21	0.57	0.04	5.74	0.64
JAK2 (allele burden)	−0.006	0.04	0.99	0.91	1.07	0.86
Headache	−3.17	1.62	0.04	0.0006	0.60	0.05
FMD	−0.35	0.15	0.71	0.49	0.90	0.02
Hemoglobin	0.19	0.09	1.21	1.06	1.55	0.03
Erythrocytes	−3.12	1.71	0.04	0.0004	0.53	0.06
Platelets	0.0002	0.004	1.00	0.99	1.01	0.96
Leukocytes	0.23	0.37	1.26	0.61	2.80	0.53
ESR	0.02	0.14	1.03	0.75	1.37	0.86
Hydroxyurea	1.95	1.49	7.01	0.52	2.3 × 10^2^	0.19

## Data Availability

The data that support the findings of this study are available from the corresponding author, P.K., upon reasonable request.

## Abbreviations

MPD	myeloproliferative disorders
ET	essential thrombocytemia
PV	polycythemia vera
PMF	primary myelofibrosis
FMD	flow-mediated dilation
